# Population pharmacokinetic modeling of Sepantronium bromide (YM155), a small molecule survivin suppressant, in patients with non-small cell lung cancer, hormone refractory prostate cancer, or unresectable stage III or IV melanoma

**DOI:** 10.1007/s10637-012-9867-x

**Published:** 2012-08-16

**Authors:** Yumiko Aoyama, Atsunori Kaibara, Akitsugu Takada, Tetsuya Nishimura, Masataka Katashima, Taiji Sawamoto

**Affiliations:** Clinical Pharmacology, Astellas Pharma Inc., 3-17-1, Hasune, Itabashi-ku, Tokyo, 174-8612 Japan

**Keywords:** Sepantronium bromide, YM155, Population pharmacokinetics, Non-small cell lung cancer, Hormone refractory prostate cancer, Unresectable stage III or IV melanoma

## Abstract

*Purpose* Population pharmacokinetics (PK) of sepantronium bromide (YM155) was characterized in patients with non-small cell lung cancer, hormone refractory prostate cancer, or unresectable stage III or IV melanoma and enrolled in one of three phase 2 studies conducted in Europe or the U.S. *Method* Sepantronium was administered as a continuous intravenous infusion (CIVI) at 4.8 mg/m^2^/day over 7 days every 21 days. Population PK analysis was performed using a linear one-compartment model involving total body clearance (CL) and volume of distribution with an inter-individual random effect on CL and a proportional residual errors to describe 578 plasma sepantronium concentrations obtained from a total of 96 patients by NONMEM Version VI. The first-order conditional estimation method with interaction was applied. *Results* The one-compartment model with one random effect on CL and two different proportional error models provided an adequate description of the data. Creatinine clearance (CL_CR_), cancer type, and alanine aminotransferase (ALT) were recognized as significant covariates of CL. CL_CR_ was the most influential covariate on sepantronium exposure and predicted to contribute to a 25 % decrease in CL for patients with moderately impaired renal function (CL_CR_ = 40 mL/min) compared to patients with normal CL_CR_. Cancer type and ALT had a smaller but nonetheless significant contribution. Other patient characteristics such as age, gender, and race were not considered as significant covariates of CL. *Conclusions* The results provide the important information for optimizing the therapeutic efficacy and minimizing the toxicity for sepantronium in cancer therapy.

## Introduction

Survivin is an apoptosis protein that has been implicated in both cell survival and the regulation of mitosis in cancer [1]. While undetectable in most normal, differentiated tissues with the exception of the placenta, testes, and rapidly dividing cells such as CD34^+^ [1–5], survivin is highly expressed in most human malignancies, including lung, melanoma, breast, and aggressive non-Hodgkin lymphoma (NHL). Studies have reported that the suppression of survivin induces tumor cell apoptosis, and also enhances sensitivity to apoptosis induced by existing anti-cancer drugs and other apoptosis stimuli [2], making it a potential target for cancer therapy. Although relationship between the suppression of survivin and tumor cell apoptosis has been well investigated, its mechanism of action remain unclear.

Sepantronium bromide (YM155, 1-(2-methoxyethyl)-2-methyl-4,9-dioxo-3-(pyrazin-2-ylmethyl)-4,9-dihydro-1*H*-naphtho[2,3-*d*]imidazolium bromide, abbreviated as sepantronium) is a small molecule survivin suppressant that was identified by cell-based high-throughput screening and lead optimization. Sepantronium selectively suppresses survivin expression to cause activation of caspases and apoptosis induction in hormone refractory prostate cancer cells and has been shown to have broad antitumor activity and induce tumor regressions in various xenograft models [6–9]. Furthermore, a time-dependent enhancement of anti-tumor activity of sepantronium was suggested via continuous infusion [8].

Phase 1 study was conducted to evaluate safety, tolerability, efficacy and pharmacokinetics (PK) of sepantronium as mono-therapy enrolling patients with advanced solid tumors or NHL [10]. Phase 2 studies were conducted to evaluate the safety, efficacy and PK of sepantronium as mono-therapy in patients with non-small cell lung cancer (NSCLC), hormone refractory prostate cancer (HRPC), or unresectable stage III or IV melanoma (MM) with modest single-agent activity [11–13]. Phase 2 studies with combination therapy have also been conducted in patients diagnosed with various types of solid tumors [14–16].

PK profile of sepantronium was evaluated in the phase 1 study by non-compartment model analysis. Mean values for the elimination half-life, the total body clearance (CL), and the distribution volume (V) of sepantronium at 4.8 mg/m^2^/day are 26.3 h, 47.7 L/h, and 1763 L, respectively, and the urinary excretion ratio of unchanged drug ranged from 18.3 % to 28.6 % of the dose [10].

The phase 1 study showed that Sepantronium was generally well tolerated, with a maximum tolerable dose (MTD) of 4.8 mg/m^2^/day when administered as continuous intravenous infusion (CIVI) for 7 days every 21 days. On the other hand, reversible elevation in serum creatinine was found as a dose limiting toxicity (DLT). One previous non-clinical toxicology study found that short-term exposure at high plasma concentrations causes nephrotoxicity [17]. The DLT is consistent to the nephrotoxicity observed in non-clinical toxicology study, and it is suggesting effective sepantronium therapy depends on an appropriate concentration window. However, little is known about which factors affect the PK of sepantronium.

Here, we characterize the effect of covariates on PK of sepantronium and evaluate the extent of covariate effect following the administration as CIVI for 7 days (168 h) using a population PK model approach by analyzing data from 3 different phase 2 studies in sepantronium mono-therapy that enrolled patients with NSCLC, HRPC, or MM.

## Methods

### Study design

The population PK database was comprised of data from three open-label, multi-center, phase 2 studies [11–13]. These phase 2 studies involved CIVI of sepantronium for 7 days (168 h) at 4.8 mg/m^2^/day every 21 days in patients with NSCLC (33 patients), HRPC (34 patients), or MM (29 patients), ranging in age from 29 to 90 years. Patients in the NSCLC study were enrolled in Europe, while those for the other two studies came from North America. Sepantronium was prepared for administration by diluting an appropriate volume of concentrated stock solution in 5 % dextrose in a light- and temperature-controlled environment. The first sepantronium dose was calculated using actual body surface area (BSA). One cycle was composed of a 7-day (168-h) administration period and a 14-day observation period (1 cycle). Doses were expressed as those of the cationic moiety of sepantronium bromide.

These studies were approved by an Ethics Committee or Institutional Review Board and were conducted in accordance with the current revision of the Declaration of Helsinki.

### Pharmacokinetic sampling

PK blood samples (6 mL/tube) were collected in sodium heparin tubes. Samples were drawn during a 7-day (168-h) CIVI, at immediately before stop of the CIVI, and 0.5–4 and 6–24 h after stop of the CIVI on cycle 1, and during a 7-day CIVI on other cycles. A total of 578 PK blood samples were obtained from 96 patients.

### Bioanalytical procedures

Plasma concentrations of sepantronium were assayed using a slightly modified version of a previously reported validated liquid chromatography tandem mass spectrometry (LC-MS/MS) method [10]. The bioanalysis was performed at PPD Central Laboratory (Richmond, VA, USA). The lower limit of quantitation was 0.05 ng/mL in plasma. Concentrations were expressed as those of the cationic moiety of sepantronium bromide. The plasma sepantronium assay was linear at least 0.05 ng/mL and had excellent inter-day precision (<18.5 % for 0.05–40.0 ng/mL sepantronium), accuracy (<7.46 % deviation from nominal concentration), and recovery (84.2 %–86.0 % for 0.15–40.0 ng/mL). No compounds were seen to interfere with the sepantronium assay.

### Data analysis

The population PK analysis was performed via nonlinear mixed-effect modeling with the NONMEM software, Version VI (ICON Development Solutions, Ellicott City, MD, USA) [18]. PDX-Pop Version 3.0 (ICON) was used to track all code patches/options. The first-order conditional estimation method with interaction option (FOCEI) in NONMEM was employed for all model runs. Plasma sepantronium concentrations that were below the lower limit of quantitation (0.05 ng/mL) or missing values were excluded from the analysis.

The final model selection was performed using various diagnostic indicators including comparisons based on the minimum objective function value (OFV), visual inspection of goodness-of-fit plots, and estimation errors for population fixed and random effect parameters. The population PK was developed in a series of steps as follows: i) base model development, ii) covariate model development, and iii) model validation.

A one-compartment model was applied to describe the plasma sepantronium concentration-versus-time profiles. The structural PK model was parameterized in terms of CL and V, as implemented using NONMEM with the ADVAN1 and TRANS2 subroutines. All inter-individual random effects were modeled exponentially (Eq. 1). An attempt was made to define a full block covariance matrix for the inter-individual random effects (Ω) when possible. A graphical assessment of goodness-of-fit plots was conducted to confirm the adequacy of the base model.

1 $$ {{\text{P}}_{\text{i}}} = {{\text{P}}_{\text{pop}}} \times { \exp }\left( {{\eta_{\text{Pi}}}} \right) $$

In Eq. 1, P_i_ is the parameter value for an individual i, P_pop_ is the typical population value of the parameter, and ηP_i_ is individual-specific inter-individual random effect for parameter P of individual i, which are assumed to be distributed as η ~ N(0, ω^2^).

For PK observations in this analysis, a residual error model was initially described by a proportional error model (Eq. 2).

2 $$ {{\text{C}}_{{{\text{obs}},{\text{ij}}}}} = {{\text{C}}_{{{\text{pred}},{\text{ij}}}}} \times \left( {{1} + {\varepsilon_{\text{ij}}}} \right) $$

In Eq. 2, C_obs,ij_ is the j-th measured observation of individual i, C_pred,ij_ is the j-th model predicted value of individual i, ε_ij_ is a random effect for measurement j of individual i and, respectively, and are each assumed to be independently distributed as ε ~ N(0, σ^2^).

### Covariate model

Individual empirical Bayesian (post-hoc) estimates of CL were obtained from the base model. Symmetry in the distributions of the post-hoc estimates of ηCL were assessed graphically. Effects of covariates on CL were typically modeled using the following equations for continuous and binary variables, respectively.

3 $$ {\text{C}}{{\text{L}}_{{{\text{pop}}|{\text{X}} = {\text{Xj}}}}} = {\text{C}}{{\text{L}}_{{{\text{pop}}|{\text{X}} = {\text{Xpop}}}}} \times {\left( {{{\text{X}}_{\text{j}}}/{{\text{X}}_{\text{pop}}}} \right)^{{\theta {\text{power}}}}} $$

4 $$ {\text{C}}{{\text{L}}_{{{\text{pop}}|{\text{Z}} = {\text{Zj}}}}} = {\text{C}}{{\text{L}}_{{{\text{pop}}|{\text{Z}} = 0}}} \times {\theta_{\text{ratio}}}{,_{\text{Zj}}} $$

In Eqs. 3 and 4, X_j_ and Z_j_ are the continuous and indicator variables for the j-th subject, respectively, and θ_power_ or θ_ratio_ are the fixed effect parameters. X_pop_ is a representative value of X in the population, which is a median or an arbitrary value close to the median. Z_j_ has a value of 1 when the j-th subject belongs to the category, otherwise it is 0. When a model contains multiple continuous and discrete covariates, the additional multiple factors were given in the forms of Eqs. 3 or 4, respectively.

The covariate exploration was performed by taking the following steps: First, a graphical assessment along with a linear regression test or *t*-test was made for screening out covariates that clearly did not exhibit any trends. In the second step, the screened covariates were subjected to a forward addition algorithm. Inclusion was determined based on the results of a likelihood ratio test with a significance level of 0.05 or better. In the final step, a backward elimination, where an exclusion of each covariate one at a time from the full model in the step 2, was examined by likelihood ratio test with a significance level of 0.01 or better to obtain the final model.

Covariates investigated using this analysis included, cycle, age, sex, race, alanine aminotransferase (ALT), aspartate aminotransferase (AST), serum creatinine, creatinine clearance (CL_CR_), measured body size (body weight [BW], BSA, and height), albumin, α_1_-acid glycoprotein (α_1_-AGP), cancer type (study), and the Eastern Cooperative Oncology Group (ECOG) performance status [19] on CL. CL_CR_ was estimated from serum creatinine concentration, age and BW using the Cockcroft-Gault equation [20] shown in Eq. 5 and estimated values were used directly for the analysis.

5 $$ {\text{C}}{{\text{L}}_{\text{CR}}}\left( {{\text{mL}}/{ \min }} \right) = \left\{ {\left[ {{14}0 - {\text{Age}}\left( {\text{years}} \right)} \right] \times \left[ {{\text{BW}}\left( {\text{kg}} \right)} \right]} \right\}/\left[ {{72} \times {\text{creatinine}}\left( {{\text{mg}}/{\text{dL}}} \right)} \right]\left\{ { \times 0.{85}\left( {\text{if female}} \right)} \right\} $$

Besides the above exploration processes, possible covariates were chosen with considering scientific interest, mechanistic plausibility, and prior knowledge to avoid unstableness of modeling due to correlation or co-linearity.

### Model validation

The reliability of the final model and parameter estimates were investigated by a nonparametric bootstrap procedure [21, 22]. A total of 300 replicate data sets were generated by random sampling with replacement. Population parameters for each data set were subsequently estimated. Empirical 95 % CIs were constructed by the 2.5th and 97.5th quartiles of population parameter estimates for each bootstrap runs with successful convergence.

### Exposure parameter

Steady-state plasma sepantronium concentration during CIVI (C_SS_) was calculated as a representative individual exposure parameter from following equation:

6 $$ {{\text{C}}_{\text{SS}}}\left( {{\text{ng}}/{\text{mL}}} \right) = {\text{Dose}}\left( {\text{mg}} \right)/\left[ {{\text{CL}}\left( {{\text{L}}/{\text{h}}} \right) \times {\text{CIVI}}\;{\text{duration}}\left( {\text{h}} \right)} \right] \times {1}000 $$

In Eq. 6, dose is individual actual dose and CL is individual post-hoc clearance from the final model.

## Results

### Analytical population and data characteristics

The analysis database was comprised of 96 patients (17 females and 79 males) contributing a total of 578 plasma sepantronium concentrations (Table 1).

**Table 1:** Numbers of patients and concentrations by study

Cancer type (Study)	Number of patients	Number of plasma sepantronium concentrations	Average number of concentrations per patients
NSCLC	33	191	5.8
HRPC	34	223	6.6
MM	29	164	5.7
Total	96	578	6.0

*NSCLC*, non-small cell lung cancer; *HRPC* hormone refractory prostate cancer; *MM* unresectable melanoma

Patient demographics at screening are presented in Table 2. For most patient characteristics except for α_1_-AGP and AST, there is no statistically significant difference among cancer types (*P* > 0.05 for age, height, serum creatinine level and CL_CR_, and *P* > 0.01 for others). Median values of α_1_-AGP in patients with MM (22 μmol/L) were lower than those in patients with NSCLC or HRPC (35 μmol/L in both). Median values of AST in HRPC (34 U/L) were higher than in other cancer (21 U/L in NSCLC and 24 U/L in MM). All HRPC patients were male and all NSCLC patients were Caucasian. ECOG performance status was Grade 0 or 1 in MM, while Grade 0, 1 or 2 in NSCLC and HRPC.

**Table 2:** Characteristics of patients included in the population PK analysis (*N* = 96)

	Median	Mean	Standard Deviation	Min	Max
Age, years	64	63	11.7	29	90
Body weight, kg	81	82	14.3	50	114
Height, m	1.76	1.74	0.08	1.53	1.92
BSA, m^2^	1.97	1.97	0.19	1.48	2.40
Albumin, g/L	38	37	4.8	22	45
α_1_-AGP, μmol/L	31	33	13.8	13	91
ALT, U/L	20	27	25.0	6	185
AST, U/L	25	30	16.3	12	92
Serum creatinine, μmol/L	88	91	23.1	53	180
CL_CR_, mL/min	79	88	31.0	31	180
	Frequency	%			
Cancer type(study)					
NSCLC	33	34.4			
HRPC	34	35.4			
MM	29	30.2			
Sex					
Female	17	17.7			
Male	79	82.3			
Race and ethnicity					
African American	4	4.2			
Caucasian	44	45.8			
Caucasian, Hispanic or Latino	8	8.3			
Caucasian, Non Hispanic or Latino	40	41.7			
ECOG performance status					
Grade 0	38	39.6			
Grade 1	53	55.2			
Grade 2	5	5.2			

*BSA* Body surface area ; *α*_*1*_ *-AGP* α_1_-acid glycoprotein; *ALT* alanine aminotransferase; *AST* aspartate aminotransferase; *CL*_*CR*_ creatinine clearance; *NSCLC* non-small cell lung cancer; *HRPC* hormone refractory prostate cancer; *MM* unresectable melanoma

**Fig. 1:**
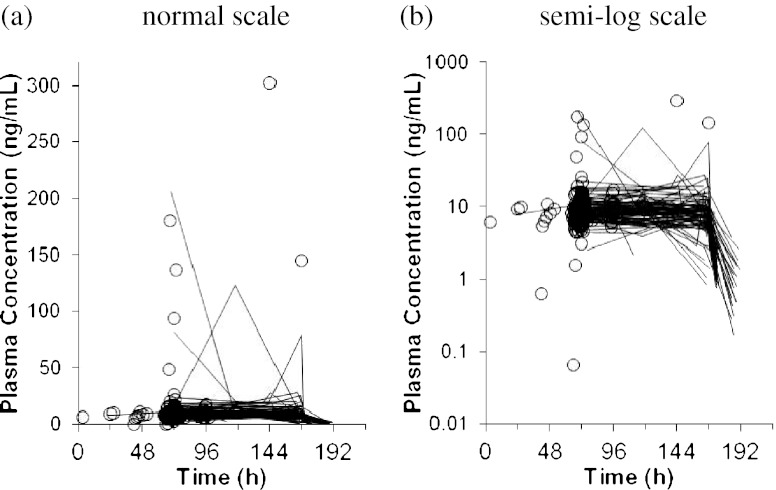
Plasma Sepantronium Concentration-Time Profiles. *Line* sequential observations taken in cycle 1, *open circle* observations in the other cycles

The plasma sepantronium concentration versus time profile is presented in Fig. 1. Plasma sepantronium concentrations were obtained at various times over a 7-day (168-h) CIVI period and over 24 h after the end of the CIVI. Some patients showed significant fluctuations in their plasma sepantronium concentrations during CIVI (Fig. 1). Since it was deemed difficult to correctly identify possible outliers with the sparse data by visual inspection or available clinical records, it was decided that no data were to be removed from the analysis data set as outliers. Instead, a separate residual error (intra-individual variability) model with a different magnitude was set for the patients who had possible outliers to allow larger residual errors. Possible outliers were then identified as follows:

7 $$ {\text{Low}}\;{\text{outlier}}:{\text{less}}\;{\text{than}}\;{\text{Q1}} - {3} \times {\text{IQR}} $$

8 $$ {\text{High}}\;{\text{outlier}}:{\text{more}}\;{\text{than}}\;{\text{Q3}} + {3} \times {\text{IQR}} $$

In Eqs. 7 and 8, Q1 and Q3 are the 1st and 3rd quartiles of plasma sepantronium concentrations taken during CIVI and IQR is the inter-quartile range of the plasma sepantronium concentrations during CIVI, i.e. Q3-Q1. In total, 11 patients with 16 plasma sepantronium concentrations that exceeded 23.13 ng/mL were identified as high outliers, while no concentrations were identified as low outliers.

### Population PK modeling

Population PK parameters derived from the base model are shown in Table 3. After evaluating various base models, inter-individual variability was assumed only in CL. The base model, i.e. one-compartment model with one random effect on CL and two different proportional error models based on having possible outliers, provided an adequate description of the data (Table 3).

**Table 3:** Population pharmacokinetic model parameter

Parameter		Final Model		Base Model	
		Estimate	Error (CV%)	Estimate	Error (CV%)
Structural PK	CL (L/h)	42.1	4.9	45.6	3.3
	V (L)	319	8.7	317	9.0
Fixed effect on CL	CL_CR_ (power)	0.425	14.5	NA	
	HRPC (ratio)	0.955	6.2	NA	
	MM (ratio)	1.24	6.3	NA	
	ALT (power)	0.124	32.2	NA	
Random effect on CL		0.0385	16.0	0.0838	17.3
Random effect on	w/o outliers	0.0934	10.2	0.0934	10.2
Cp (Residual error)	with outliers	31.6	33.9	28.6	42.0
OFV		1985.227		2043.738	

*CV* coefficient of variation; *CL*_*CR*_ creatinine clearance; *HRPC* hormone refractory prostate cancer; *MM* unresectable melanoma; *ALT* alanine aminotransferase; *OFV* objective function value

As a result of the preliminary screening by linear regressions and one-way ANOVA, age, α_1_-AGP, albumin, ALT, body surface area, BW, CL_CR_, serum creatinine, cancer type, and ECOG performance status were selected as potential covariates. The covariate exploration in the forward addition step revealed CL_CR_, cancer type and ALT are the potential covariates on CL. CL_CR_ was found to be the most influential as the addition of CL_CR_ caused a decrease in OFV of over −31 points. Cancer type and ALT had also a significant effect on CL (a decrease in OFV was −19 and −8, respectively). As the final step, the three potential covariates were tested using the backward elimination algorithm. As a result, the significance of all the covariates was confirmed.

Based on the final model including the fixed effects of CL_CR_, cancer type, and ALT, individual CL (CL_j_) was expressed as follows:

9 $$ {\text{C}}{{\text{L}}_{\text{j}}} = {42}.{1} \times {\left( {{\text{C}}{{\text{L}}_{{{\text{CR}},{\text{j}}}}}/{79}.{22}} \right)^{{0.{425}}}} \times {\left( {{\text{AL}}{{\text{T}}_{\text{j}}}/{19}.0} \right)^{{0.{124}}}} \times 0.{95}{{5}^{{({\text{if HRPC}})}}} \times {1}.{2}{{4}^{{({\text{if MM}})}}} $$

**Fig. 2:**
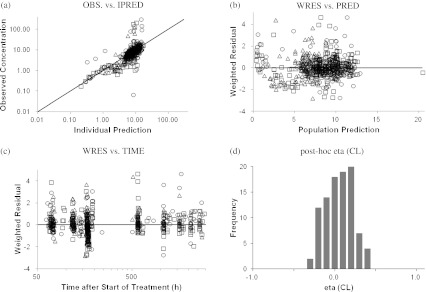
Diagnostic Plots (Final Model). Open circle; non-small cell lung cancer, open square; hormone refractory prostate cancer, open triangle; unresectable stage III or IV melanoma

The parameter estimates of the final population PK model are also shown in Table 3. The final model resulted in an improvement in the goodness-of-fit criteria, compared with the base model. The estimation errors of the estimates were adequately low in general. The inter-individual variances for CL was 0.0385 (percentage coefficient of variation [CV%], 19.6 %) in the final model. The inter-individual variability for CL was reduced in the final model, when compared with the base model (28.9 %, 0.0838 in variance) variance estimate (Table 3). Figure 2 shows a diagnostic plot of the final PK model. The weighted residuals (WRES) over PRED and TIME were distributed around 0, suggesting no systematic bias in the model fitting. Plots of individual post-hoc estimates from the final model versus the significant covariates revealed no remaining trends. Distributions of inter-individual random effects are centered at the expected value of zero, as indicated by the eta-bar estimates included in the NONMEM output (data not shown).

The final model was used to simulate C_SS_ of sepantronium. The magnitude of the covariate effects of CL_CR_ on CL was predicted to contribute a 25 % decrease in CL, which would consequently lead to a 34 % increase in C_SS_ in patients with moderate impaired renal function (CL_CR_ =40 mL/min).

### Model validation

The final model was subjected to a bootstrap analysis. A total of 299 of 300 runs were successfully minimized. The bootstrap means and confidence intervals from 299 successful runs out of 300 were well consistent with the parameter estimates of the final model as shown in Table 4, which indicates robustness of the final model.

**Table 4:** Bootstrap estimates of final model

Parameter		Bootstrap (*n* = 299)		Final Model	
		estimate	95 % CI	estimate	95 % CI
Structural PK	CL (L/h)	42.1	38.2–46.6	42.1	38.1–46.1
	V (L)	320	268–379	319	265–373
Fixed effect on CL	CL_CR_ (power)	0.424	0.306–0.524	0.425	0.304–0.546
	HRPC (ratio)	0.958	0.825–1.08	0.955	0.838–1.07
	MM (ratio)	1.24	1.10–1.41	1.24	1.09–1.39
	ALT (power)	0.125	0.042–0.218	0.124	0.046–0.202
Random effect on CL		0.036	0.025–0.047	0.0385	0.026–0.051
Random effect on	w/o outliers	0.092	0.076–0.111	0.0934	0.075–0.112
Cp (residual error)	with outliers	32.4	12.1–57.3	31.6	10.6–52.6

*CI* confidence interval; *CL*_*CR*_ creatinine clearance; *HRPC* hormone refractory prostate cancer; *MM* unresectable melanoma; *ALT* alanine aminotransferase

### Exposure—safety relationship

**Fig. 3:**
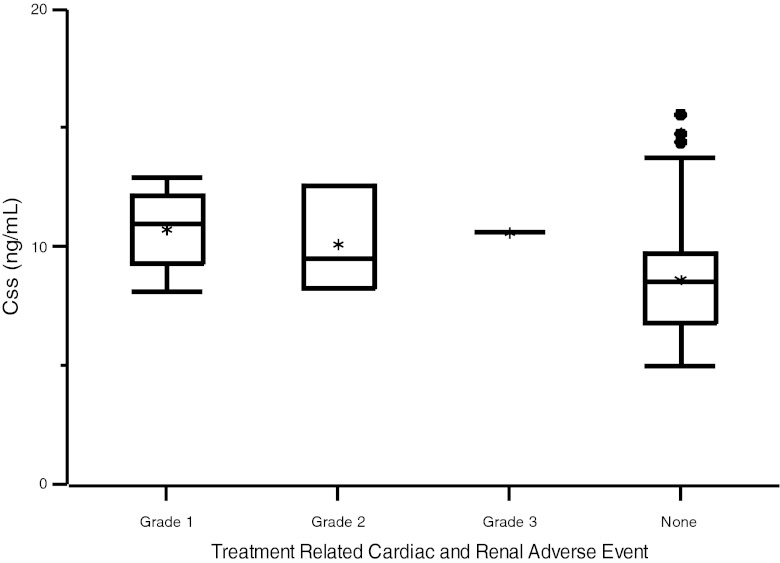
Relationship between steady state concentration of YM155 and treatment related cardiac and renal adverse event. Grade 1, *n* = 4: Grade 2, *n* = 3: Grade 3, *n* = 1: None, patients without treatment related cardiac or renal adverse event, *n* = 88. Star represents a mean value: box represents a range of 50% interval: a bar in each box represents a median value: bar under the box represents a 25 percentile: bar over the box represents a 75 percentile: fixed circle represents outlier

Relationship between simulated C_SS_ and treatment related cardiac and renal adverse event was presented in Fig. 3. There was no obvious relationship between C_SS_ and these adverse events.

## Discussion

We present here a population PK modeling of sepantronium based on 578 samples obtained during CIVI or up to 24 h after stop of CIVI from a total of 96 cancer patients suffering from various types of advanced solid tumors. Dose regimens were CIVI for 7 days at 4.8 mg/m^2^/day. The final model provided population mean estimates of 42.1 L/h and 319 L for CL and V, respectively. The estimate of CL was in line with the previous results of 27.6–47.7 L/h obtained by phase 1 study carried out in advanced solid tumors or NHL patients [10].

In a preliminary investigation, it was suggested that individual Bayes estimates were strongly dependent on whether or not patients had any measurable plasma concentration data after end of infusion when a two-compartment model was applied (data not shown). The individual Bayes estimate of V still showed a weak relationship with elapsed time after end of infusion of the last measurable concentration data in cycle 1 even when a one-compartment model was applied, suggesting the difficulty in separately estimating the inter-patient variability in V from that in CL. To focus on estimating reliable individual exposure parameter especially during infusion, a simple one-compartment model was chosen as a basic PK model. Nevertheless, the use of fixed V enabled us to model the data and obtain good estimates for CL, which is valuable in the regulation of sepantronium therapy.

In a few patients, fluctuations with very high C_SS_ were observed during CIVI and concentrations could vary more than 50-fold from one value to another during CIVI, where almost flat C_SS_-time profile was expected theoretically (Fig. 1). Indeed, on further analysis, 16 plasma sepantronium concentrations obtained from 11 out of 96 patients were extracted as outliers. One of possible reasons was accidental contamination at blood sampling or bioanalytical procedure. However no errors implying such contamination were found. Furthermore, these outliers could not be explained by any patient characteristics or other factors, as they were observed in all cancer types (*n* = 5 in NSCLC, *n* = 3 in HRPC, and *n* = 3 in MM) and all cycles in which blood samples were obtained (cycles 1–6). Because this is the first population PK analysis for sepantronium, it seemed inappropriate to exclude the high outliers without any firm reasons. The final model with two different residual errors reasonably handled the outliers and enabled to provide reliable parameter estimates without data exclusions.

The covariate exploration suggests that CL_CR_, cancer type and ALT were recognized as significant covariates of sepantronium CL. As urinary excretion ratio of unchanged drug is 18.3 %–28.6 % of the dose [10], therefore, renal excretion is obviously one of major elimination routes for sepantronium, it deemed quite reasonable that CL_CR_ was selected as the most influential covariate in the analysis.

Although ALT level was selected as a significant covariate of CL in the analysis, the effect of ALT was expected minimal effect on the CL of sepantronium because the exponent of the fixed effect model for ALT (power functions on CL) was close to 0 (i.e. 0.124). However, significant increase in sepantronium exposure due to severe hepatic impairment could not be fully ruled out because the patient population in the analysis did not have evaluable numbers of patients with high ALT beyond the upper limit of normal (40 U/L). Further, the final model suggests that patients with MM have slightly higher CL than those with other solid tumors such as NCSLC or HRPC (Table 3). However, because each study had patients with different cancer type, it could not be concluded which factor has a potential effect on CL, cancer type or differences in the other conditions between studies. Inclusion of these factors such as ALT and cancer type significantly improved the population PK models based on the likelihood ratio test and subsequently decreased the inter-individual variability in CL of sepantronium: however, the clinical relevancy of effects of cancer type and ALT on CL was thought to be negligible at least among the patient populations studied.

Age, gender, and race were not selected as significant covariates on CL of sepantronium in the analysis. We also examined whether or not the concentrations of specific proteins contributed to the variability in CL of sepantronium, but did not detect any significant associations with albumin or α_1_-AGP concentrations. These results are consistent with a relatively low in vitro human plasma protein binding ratio of sepantronium (18 %–20 %) (unpublished data).

Previous non-clinical studies have found that sepantronium exerts time-dependent antitumor activity with a continuous infusion and that short-term exposure at high plasma sepantronium concentrations can cause cardiac toxicity and nephrotoxicity [8, 17]. Although sepantronium is generally safe at 7-day CIVI of 4.8 mg/m^2^/day as previously reported [10–13, 17], it is anticipated that outrageously high plasma concentration of sepantronium observed in these studies could cause adverse event related to exposure dependent cardiac and renal toxicity of sepantronium found in non-clinical studies. Nine Grade 1 or more cardiac and renal adverse events probably or possibly related to the study drug were observed in 8 of 96 patients. There was no clear relationship between C_SS_ and these adverse events as shown in Fig. 3. Grade 3 or 4 adverse events related to the study drug recorded in 11 patients with providing transient high plasma sepantronium concentrations (high outliers) were grade 3 arrhythmia (*n* = 1), hypokalaemia (*n* = 1), muscle weakness (*n* = 1), thrombocytopenia (*n* = 1), hyponatraemia (*n* = 1), and grade 4 fatigue (*n* = 1). Grade 1 tachycardia (*n* = 1) and grade 3 arrhythmia (*n* = 1) were recorded as cardiac or renal adverse events related to the study drug in patients with high C_SS_ and/or outlier concentrations. However, the high C_SS_ or outliers seemed not to associate with the cause of the events because these events were also observed in patients who had lower C_SS_. The fact suggests that therapeutic drug monitoring is not necessary for safe use of sepantronium.

In conclusion, CL_CR_ was the most influential covariate on sepantronium exposure and predicted to contribute to a 25 % decrease in CL for patients with moderately impaired renal function (CL_CR_ = 40 mL/min) compared to patients with normal CL_CR_, with cancer type and ALT having a smaller but nonetheless significant contribution. Other patient characteristics such as age, gender, and race were not considered as significant covariates of CL. The analysis provides the important information for optimizing the therapeutic efficacy and minimizing the toxicity for sepantronium in cancer therapy.
